# Autopsy of Leah Smith, the Mulatto Woman, Whose Thigh Was Amputated, 15th November, 1837, for Fungus Hæmatodes*Reported in the Medical Examiner, No. 6, p. 101.

**Published:** 1838-09-26

**Authors:** 


### CLINICAL REPORTS.

### PENNSYLVANIA HOSPITAL.

*Autopsy of Leah Smith, the mulatto woman, whose thigh was amputated, 15th November, 1837, for Fungus Hæmatodes.**

After the discharge of this woman from the hospital, she remained under the care of Dr. Wallace. She continued to enjoy good health, menstruated regularly until she became pregnant. On the 8th of August, she complained of severe pain in the right side, with dyspnœa; dyspnœa continued to increase, and slight hæmoptysis came on; and she sank suddenly on the 11th of September.

*Autopsy, September 11th*, sixteen hours after death. Present, Drs. Morris, Smith, and Biddle. No decomposition. Embonpoint. Rigidity of the joints. A considerable protuberance presented itself in the abdomen, the integuments of which were not, however, very tense. Tympanitis.

*Abdomen.*—Peritoneum healthy. The uterus contained a female child, apparently of six months. The uterus, ovaries, and placenta, were all healthy. The liver was enlarged, of a reddish brown colour, uniform throughout, and the upper half of the right lobe considerably softened—otherwise normal. The spleen was of a natural size and consistence. The stomach was empty; its mucous membrane offered, in the great cul-de-sac, a bright red punctillated injection; elsewhere, it was of a light grayish colour, and mammelated, of good consistence. The mucous membrane of the intestines offered nothing abnomal. Kidneys healthy.

*Thorax.*—Strong adhesions existed throughout the pleuræ of both sides. The right lung presented exteriorly an irregularly knotted appearance, being studded with projecting masses, of various sizes, from that of a pea to that of a pullet's egg, all covered by the pleura, and some embedded in the substance of the lung. Upon cutting into the largest tumour, of a dark external colour, it was found to be composed of an internal nucleus of white, *brain-like* matter, breaking down at the touch of the scalpel, surrounded by a mass of dark grumous blood. Upon cutting into the lung, its whole substance appeared converted into a congeries of similar masses, except in the lower lobe, which was not totally disorganized. The left lung presented externally a similar appearance to the right. Its substance was infiltrated with the like encephaloid matter, and contained a mass of dark coagulated blood, of the size of an egg, in a cyst, lined with a hard, white, resisting false membrane. No tuberculous matter was observed on either lung. The heart was normal. The stump of the thigh was examined, and presented no trace of encephaloid matter. The end of the bone was smoothly rounded off.

* Reported in the Medical Examiner, No. 6, p. 101.

